# Olfactory Susceptive Difference in Gregarious and Solitary Locusts

**DOI:** 10.3390/insects17030330

**Published:** 2026-03-18

**Authors:** Weichan Cui, Dafeng Chen, Liushu Dong, Xianhui Wang

**Affiliations:** 1 State Key Laboratory of Animal Biodiversity Conservation and Integrated Pest Management, Institute of Zoology, Chinese Academy of Sciences, Beijing 100101, China; cuiweichan@bjfu.edu.cn (W.C.); chendf910@126.com (D.C.); dongliushu@ioz.ac.cn (L.D.); 2 School of Grassland Science, Beijing Forestry University, Beijing 100083, China; 3 Key Laboratory for Warm Temperate Forest Ecosystem Conservation and Restoration of National Forestry and Grassland Administration, College of Forestry, Shandong Agricultural University, Tai’an 271018, China

**Keywords:** peripheral olfactory systems, antennal sensilla, electrophysiological response, odorant receptors, phenotypic plasticity

## Abstract

The migratory locust, *Locusta migratoria*, possesses a highly specialized olfactory system that exhibits remarkable density-dependent plasticity, which plays a crucial role in the formation of large aggregations and the resulting severe crop damage. However, the mechanisms by which population density influences phase-related plasticity in olfactory perception remain largely unexplored. Here, we conducted a comprehensive, multi-level comparison of the peripheral olfactory system between solitary and gregarious locusts. We found that solitary male locusts display the highest total number of antennal sensilla, with basiconica sensilla being the most abundant and particularly prominent in this group. At the physiological level, solitary males also displayed the greatest overall sensitivity in their electroantennogram (EAG) responses to volatile compounds highly specific to both phase and sex. At the molecular level, solitary males exhibited a significant upregulation of Or genes across all sex-phase combinations. These findings illuminate the intricate adaptation strategies of the insect peripheral olfactory system in response to environmental changes.

## Simple Summary

The migratory locust, *Locusta migratoria*, is a devastating agricultural pest that forms destructive swarms. Its olfactory system exhibits plasticity in response to population density, playing a key role in aggregation and large-scale crop damage. In this study, we performed a comprehensive, multi-level comparison of the peripheral olfactory system between solitary and gregarious locusts, exploring the specific mechanisms underlying changes in the peripheral olfactory system of locusts during density-dependent phase transitions. Our findings provide insights into the biological basis of locust swarm formation and the adaptive strategies of locust peripheral olfactory systems to changing environments.

## 1. Introduction

Locusts exhibit extreme density-dependent phase polyphenism, enabling the formation of dense migrating swarms under certain circumstances [1,2]. The formation and maintenance of this large-scale aggregation behavior highly depend on chemical communication among locusts and their precise perception of environmental chemical cues [3,4]. A comprehensive understanding of how the locust olfactory system perceives and responds to environmental cues, particularly population density changes, is crucial for elucidating the mechanisms underlying their gregariousness and outbreak formation.

Olfaction serves as a critical sensory modality for insects, mediating essential behaviors including foraging, mate location, and habitat selection [5,6]. In locusts, the olfactory system exhibits remarkable density-dependent plasticity that underlies phase polyphenism [4]. At the chemical level, significant differences in volatile profiles exist between gregarious and solitary phases in *Locusta migratoria,* which regulate the species-specific behavioral responses of the locust [7]. Phenylacetonitrile (PAN) concentrations were significantly higher in gregarious individuals than in solitary locusts and facilitates an antipredator defense by acting as an olfactory aposematic signal through predation assays [8]. Furthermore, 4-vinylanisole (4VA) previously identified as an aggregation pheromone through behavioral assays [3,9]. Conversely, dibutyl phthalate (DBP) is more abundant in solitary locusts, which has been demonstrated to function as a sex pheromone at low densities based on behavioral attraction tests [10]. At the physiological level, antennal electrophysiological responses demonstrate phase- and sex-specific olfactory sensitivity in *Schistocerca gregaria* [11,12], with comparable sex-specific EAG responses to body volatiles documented in gregarious *L. migratoria* [13]. At the molecular level, transcriptomic analyses have revealed phase-specific transcriptional regulation of chemosensory genes, including differentially expressed olfactory receptors between antennae and palps [14,15], reflecting adaptive modifications at the sensory input level. Collectively, these findings indicate that the locust olfactory system possesses high density-dependent plasticity, capable of adjusting its perception abilities and behavioral responses under different population density conditions.

The peripheral olfactory system serves as the primary gateway for insects to perceive chemical information and its plasticity is crucial for environmental adaptation. Antennal sensilla directly affect the olfactory sensitivity of insects, as the basic structures for insect olfactory perception [16,17]. Research indicates that the distribution, size, and quantity of antennal sensilla exhibit significant differences among species, which reflect the evolutionary pressures and adaptations experienced [18,19]. In species that rely on chemical signals for mate attraction, the antenna and sensilla typically display pronounced sexual dimorphism [20,21,22]. Furthermore, the diversity and abundance of antennal sensilla are crucial for insects to locate and select host plants, as demonstrated in species such as the longhorn beetle [23] and ladybird beetles [24]. In the fruit fly *Anastrepha ludens*, studies on the morphology and ultrastructure of its antennal sensilla have revealed that different types of sensilla participate in perceiving various environmental information, including odors, temperature, humidity, and motion [17]. Early ultrastructural studies established the foundation for understanding antennal sensilla diversity in Orthoptera, with detailed categorization of sensilla type [25,26]. Notably, there are significant differences in the sensillum numbers between the nymphs, adults, and phases of *S. gregaria*, as well as differences due to different dietary effects on sensilla abundance in grasshoppers [27,28]. Collectively, these studies suggest that the quantity and types of antennal sensilla serve as important indicators for evaluating and understanding insect olfactory function. However, current understanding of the specific mechanisms underlying changes in the peripheral olfactory system of locusts during density-dependent phase transitions remain limited.

This study compared the peripheral olfactory systems of gregarious and solitary locusts, *L. migratoria*. By integrating (i) high-resolution scanning electron microscopy to quantify antennal sensilla types and densities, (ii) electroantennography (EAG) recordings to assess peripheral olfactory sensitivity to volatile compounds highly specific to both phase and sex, and (iii) antennal transcriptomics to profile expression of the Ors repertoire. The phase- and sex-specific differences would reveal how ecological contexts interact to reshape the peripheral olfactory system.

## 2. Materials and Methods

### 2.1. Insect Rearing

Locusts were reared at the Institute of Zoology, Chinese Academy of Sciences. Gregarious locusts were kept in cages measuring 30 cm × 30 cm × 30 cm with good ventilation and suitable lighting, and the density was controlled at 600–700 individuals per cage. Gregarious locusts were reared in the laboratory for at least three generations. Solitary locusts were individually housed in 10 cm × 10 cm × 25 cm metal boxes from hatching to adult for at least three generations. The rearing conditions were as follows: temperature 30 ± 2 °C, photoperiod L14:D10, relative humidity 60 ± 5%, and fed with fresh wheat seedlings and bran. Unmated adult male and female locusts were used in the experiments.

### 2.2. Scanning Electron Microscopy (SEM)

A total of eight adult locusts (four males and four females) were used for SEM analysis. Each antenna was excised from the head with fine forceps, then immersed in 2.5% glutaraldehyde and fixed at 4 °C for 24 h. After primary fixation, the specimens were post-fixed in 1% osmium tetroxide buffered with 0.1 M sodium cacodylate (pH 7.2). Dehydration was carried out at 4 °C through an ascending acetone series (30%, 50%, 70%, 80%, 90%, 95%, and 100%), with two changes of 15 min each per grade. The dehydrated samples were subsequently dried by immersion in tetramethylsilane (TMS) for 5–10 min, which was repeated twice. After drying, the antennae were mounted on aluminum stubs with conductive carbon tape, sputter-coated with gold–palladium in a fine coat Ion sputter JFC 1100 (JEOL, Tokyo, Japan), and examined under a JSM-6360 scanning electron microscope (JEOL, Japan).

### 2.3. Total RNA Isolation, Transcriptome Profiling, and qPCR Validation

Antennae were collected from adult locusts and immediately frozen in liquid nitrogen. Total RNA was extracted with TRIzol Reagent (Invitrogen, Carlsbad, CA, USA) following the manufacturer’s instructions. Quantity and purity were assessed on a NanoDrop ND-1000 spectrophotometer (Thermo, Wilmington, WI, USA) (260/280 nm ratio of 1.8–2.0). Purity was measured by the 260/280 ratio, and the range of 1.8 to 2.0, indicating the absence of exogenous contamination in the RNA sample. First-strand cDNA for gene-specific assays was synthesized from 2 µg DNase I-treated RNA with M-MLV reverse transcriptase (Promega, Wisconsin, WI, USA) and oligo (dT)18 primers.

RNA-seq libraries were constructed from 2 µg of antennal RNA (three independent replicates per condition) using the TruSeq RNA Sample Prep Kit v2 (Illumina Inc., San Diego, CA, USA). Paired-end 150 bp reads were generated on an Illumina HiSeq 2000 platform (Illumina, Inc., San Diego, CA, USA). After adapter trimming and quality filtering with Trimmomatic (default settings), clean reads were mapped to the *L. migratoria* reference genome (v2.4.1) with TopHat2. Uniquely mapped reads were counted by HTSeq and normalized to fragments per kilobase of transcript per million mapped reads (FPKM). Differentially expressed genes (DEGs) were identified with DESeq2 (log2FC ≥ 1, FDR < 0.05) and subjected to Gene Ontology enrichment analysis using GOstat with the entire annotated gene set as background.

For quantitative RT-PCR, 2 µg RNA was reverse-transcribed with Moloney Murine Leukemia Virus Reverse Transcriptase (Promega, cat. M1701). Reactions were run in technical triplicate on a LightCycler 480 system (Roche Diagnostics, Meylan, France) with SYBR Green I Master Mix (Roche, cat. 04707516001). Melting curve analysis confirmed single amplicons. Expression values were normalized to the ribosomal protein RP49 gene and calculated by the 2^−ΔCt^ method. Primer sequences are provided in Supplementary Materials, Table S1.

### 2.4. Electroantennography (EAG)

We tested the EAG response to the 16 components and solvent was used as the control. Each compound was added onto filter paper (0.2 cm × 1 cm) at 10 μL in a Pasteur tube, resulting in final loading capacities of 0.1 ng to 1 mg for each chemical. EAG values were recorded according to the standard method [3,10]. Briefly, one antenna (on the left side of the head from the dorsal view) per locust was cut at the base with a blade, and the tips of the antenna were removed to approximately 1 mm in length. Conductive gel was applied to the ends of the electrodes, and the prepared antennae were connected to the electrodes. After the circuit was connected and the software was ready for recording, the airflow pump was turned on to ensure that the intake bottle contained distilled water. After the baseline stabilized, the experiment began. The antennae were first stimulated with a Pasteur pipette containing solvent, followed by a 1 min pause. Then, the antennae were stimulated with a Pasteur pipette containing the corresponding odor, followed by another 1 min pause. After all odor stimuli were applied, a new locust antenna was used for the next round of stimulation. The order of odor stimulation was randomized. Half-maximal effective concentrations (EC50) with 95% confidence intervals were estimated using the nonlinear curve fit regression function in GraphPad Prism. An IDAC-2 amplifier (Syntech, Kirchzarten, Germany) was coupled to a computer installed with EAGPro2.0 (Syntech, Germany) for data acquisition, recording, and analysis. For statistical analysis, EAG amplitudes were calculated by subtracting the solvent response.

### 2.5. Data Analysis and Statistics

We used the Kolmogorov–Smirnov test to assess normal distribution. Two-way ANOVA was performed to examine the effects of sexes (male/female) and phases (gregarious/solitary) on number of sensilla, EAG responses, and relative expression of Ors. The interaction between sexes and phases was examined first. If the interaction was significant, simple effects analyses were conducted using Student’s *t*-tests to compare gregarious vs solitary phases within each sex, and sex differences within each phase. If the interaction was not significant, main effects were interpreted directly. Significant main effects were followed up with Student’s *t*-tests for pairwise comparisons. For non-normally distributed data, Mann–Whitney U tests were performed for simple effects analysis. All statistical analyses were conducted using GraphPad Prism 9 software (GraphPad Software, San Diego, CA, USA) and SPSS statistics 27. Statistical significance was defined as *p* < 0.05.

## 3. Results

### 3.1. Solitary Male Locusts Possess the Highest Number of Antennal Sensilla

**Figure 1:**
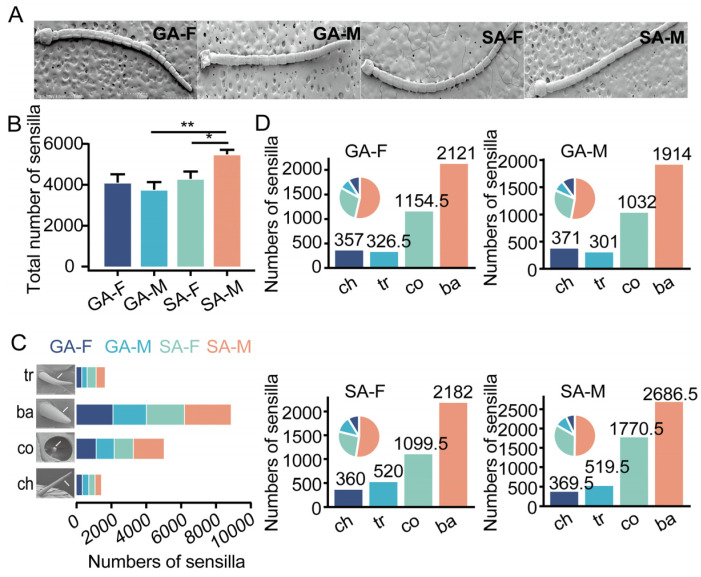
The number of different types of sensilla on locusts in various phases and sexes. (**A**) Scanning electron micrographs (SEM) of antennae from GF, GM, SF, and SM (gregarious females, GF; gregarious males, GM; solitary females, SF; solitary males, SM). Scale bar = 1 mm (S-4800, 2.0 kV, 8.0 mm × 30 SEM). (**B**) Total number of antennal sensilla in GF, GM, SF, and SM. Data are presented as the mean ± s.e.m. *p* values are determined by two-tailed unpaired *t* test (* p < 0.05, ** *p* < 0.01). (**C**) Number of different types of sensilla on the antennae of GF, GM, SF, and SM. (**D**) Number and proportion of sensilla for each type on the antennae of GF, GM, SF, and SM. (Trichoid sensilla, Tr, 5 µm; Coeloconica sensilla, Co, 3 µm; Basiconica sensilla, Ba, 4 µm; Chaetica sensilla Ch, 10 µm; n = 3–4).

We systematically quantified the number of antennal sensilla in locust adults using SEM, considering different sexes (female vs. male) and phases (gregarious vs. solitary) (gregarious females, GF; gregarious males, GM; solitary females, SF; solitary males, SM) (Figure 1A). The numbers of antennal sensilla showed significant differences in the interaction between sexes and phases (two-way ANOVA, F_1,11_ = 4.91, *p* = 0.049), while neither the main effect of sex (F_1,11_ = 0.47, *p* = 0.51) nor phase (F_1,11_= 0.15, *p* = 0.71) was significant. Simple effects analysis showed that there was no significant difference in the total number of sensilla between GF and GM, with both sexes having approximately 4000 sensilla. For solitary locusts, males had a significantly higher total number of sensilla than females with approximately 25% more. For the comparison between two phases, SM had a significantly higher total number of sensilla than GM, whereas there was not a significant difference in females (Figure 1B).

In addition, we separately quantified the number of different types of sensilla in both sexes and both phases of locusts. The results showed that basiconica sensilla were the most abundant, followed by coeloconica, trichoid, and chaetica sensilla in locusts. SM exhibited the highest number of basiconica sensilla across all combinations. In contrast, chaetica and trichoid sensilla were markedly less numerous, each totaling fewer than 500 (Figure 1C,D). In summary, SM had the highest total number of sensilla, with basiconica sensilla being the predominant component of locust antennal sensilla.

### 3.2. Solitary Male Locusts Exhibit Stronger Electroantennographic (EAG) Responses to Volatiles

To evaluate the olfactory perception sensitivity across phases and sexes, we measured the EAG responses of locusts to 16 volatile compounds derived from locust feces, host plants and the locust body. The results demonstrated that these volatiles elicited significant EAG responses with distinct response patterns in both sexes and both phases of the locust (Table S2). Dibutyl phthalate exhibited a significant interaction effect (sex × phase: F_1,47_ = 59.07, *p* < 0.001), with SM (mean peak amplitude = 0.20 mV) showing a significant response compared to GM (mean peak amplitude = 0.02 mV), whereas females showed no phase-dependent response between SF (mean peak amplitude = 0.01 mV) and GF (mean peak amplitude = 0.01 mV). Four compounds (4-vinylanisole, anisole, benzaldehyde, and phenol) exhibited parallel main effects of both sex and phase, with solitary locusts showing elevated responses compared to gregarious individuals (sex: F_1,49_ =7.21, *p =* 0.010, phase: F_1,49_ = 5.46, *p =* 0.024; sex: F_1,48_ = 8.47, *p =* 0.005, phase: F_1,48_ = 7.00, *p* = 0.011; sex: F_1,49_ = 9.91, *p =* 0.003, phase: F_1,49_ =7.97, *p =* 0.007; sex: F_1,48_ = 18.12, *p <* 0.001, phase: F_1,48_ = 21.10, *p <* 0.001). Specifically, the comparison between SM and GM revealed that anisole, benzaldehyde, and phenol elicited significantly stronger responses in SM (mean peak amplitudes: 1.2 mV, 1.0 mV, and 0.5 mV, respectively) than in GM. Conversely, pentanoic acid displayed phase-only effects with a unique reversed pattern, where gregarious locusts exhibited higher responses than solitary individuals (phase: F_1,49_ = 11.22, *p =* 0.002).

Seven compounds exhibited sex-specific effects exclusively. Comparison between SM and SF showed that phenethyl alcohol elicited distinct EAG response patterns (mean peak amplitude = 0.8 mV). A significant difference in response to phenethyl alcohol was also observed between GM and GF (mean peak amplitude = 0.8 mV, sex: F_1,48_ = 13.88, *p* < 0.001). Other compounds showing sex-only effects included guaiacol (F_1,48_ = 12.04, *p* = 0.001), phenylacetonitrile (F_1,48_ = 10.39, *p* = 0.002), veratrole (F_1,50_ = 16.12, *p* < 0.001), E-2-hexenol (F_1,50_ = 9.47, *p* = 0.003), 2-penten-1-ol (F_1,50_ = 7.63, *p* = 0.008), and cis-3-hexen-1-ol (F_1,50_ = 7.34, *p =* 0.009), with the latter also showing phase-dependent differences between SF and GF (mean peak amplitude = 1.5 mV). In contrast, three compounds elicited no significant differential responses across sexes or phases.

**Figure 2:**
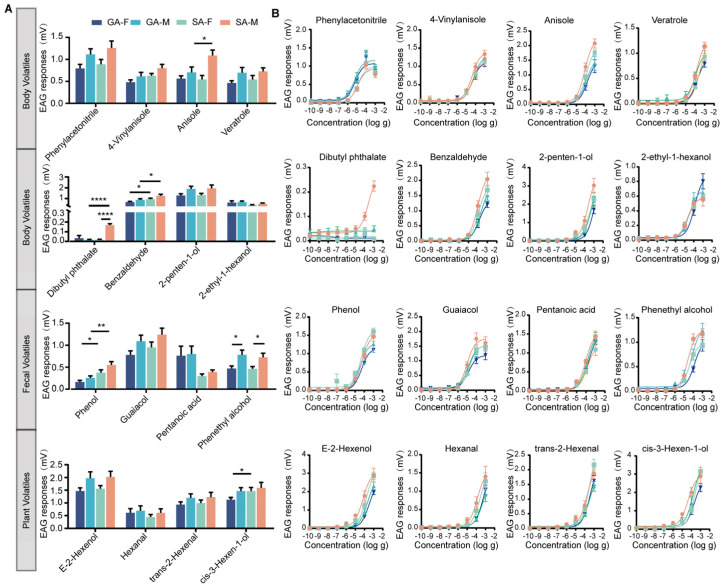
EAG responses of gregarious and solitary locusts to volatiles. (**A**) EAG responses of GF, GM, SF, and SM antennae to volatiles released from fecal matter, host-plants, and body. n_GF_ = 14, n_GM_ = 12, n_SF_ = 14, n_SM_ = 13. Data are presented as the mean ± s.e.m. *p* values refer to two-way ANOVA and Student’s *t*-tests or Mann–Whitney U tests (* *p* < 0.05, ** *p* < 0.01, **** *p* < 0.0001). (**B**) EAG dose responses of male and female antennae in both phases to synthetic compounds ranging from 0.1 ng to 1 mg. n_GF_ = 8–10, n_GM_ = 7–10, n_SF_ = 9–13, n_SM_ = 10–13 (Data are presented as the mean ± s.e.m).

We further examined the EAG dose responses of locusts to 16 compounds. The results indicated that GM and GF were sensitive only to two compounds, 4VA and PAN, with EC50 values of 5.83 × 10^−5^ mV and 4.50 × 10^−5^ mV, respectively. SM exhibited the highest sensitivity to six compounds: anisole, guaiacol, cis-3-hexen-1-ol, benzaldehyde, valeric acid, and trans-2-hexenal, with EC50 values of 8.23 × 10^−5^ mV, 9.62 × 10^−6^ mV, 4.26 × 10^−5^ mV, 9.45 × 10^−5^ mV, 9.34 × 10^−5^ mV, and 6.36 × 10^−5^ mV, respectively. These EC50 values were significantly lower than those of GM, GF, and SF, indicating that its peripheral olfactory system has a lower detection threshold and higher sensitivity to these compounds. However, no significant differences were observed in EC50 values for the remaining compounds across different phases and sexes (Figure 2B).

### 3.3. Transcriptome Analysis of Antennae Between Gregarious and Solitary Locusts

Using the Illumina HiSeq 2000 platform, we sequenced the cDNA libraries of antennae from GF, GM, SF, and SM (antennae from gregarious females, GA-F; antennae from gregarious males, GA-M; antennae from solitary females, SA-F; antennae from solitary males, SA-M) (Table S3). We utilized the fragments per kilobase of exon per million fragments mapped (FPKM) value to evaluate the expression levels of all antennal olfactory genes in female and male locusts. Cluster analysis of gene expression levels showed that samples from the same period and sex clustered well together (Figure S1).

**Figure 3:**
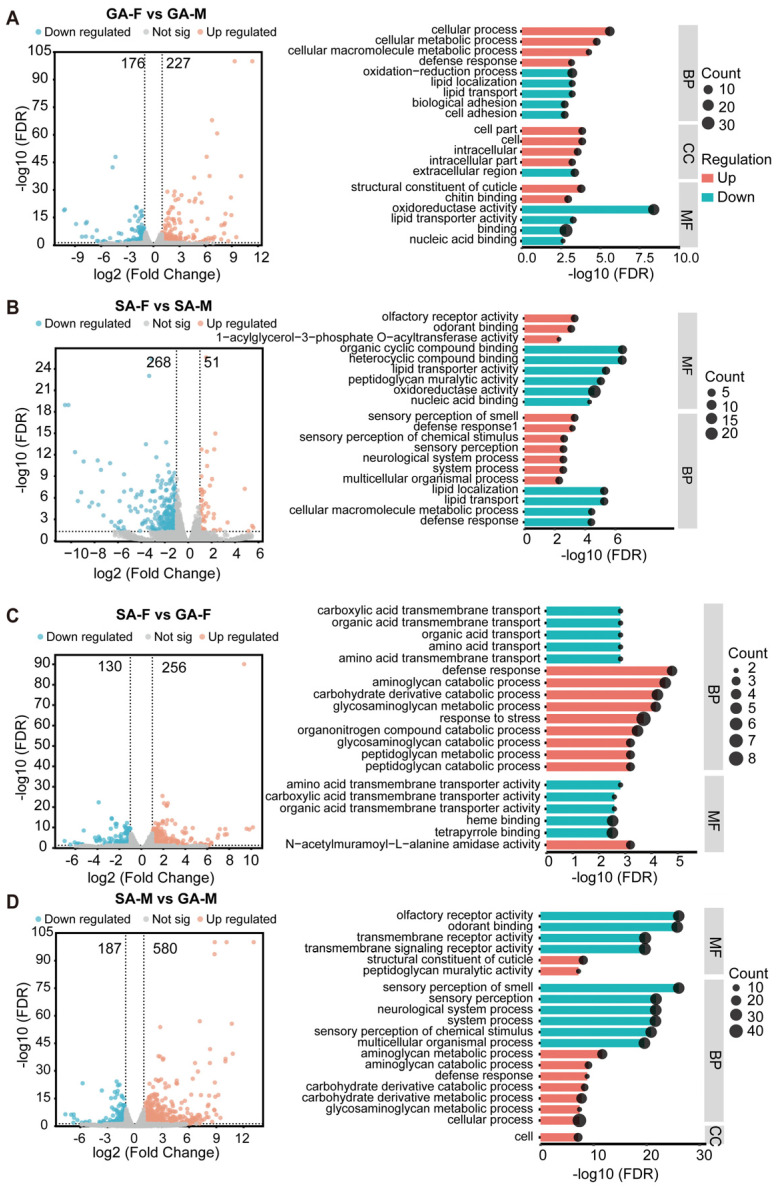
Comparison of gene expression patterns between gregarious and solitary locusts of both sexes. (**A**) Statistics on the number of differential genes and GO pathway enrichment analysis in the GA-F vs. GA-M group. (**B**) Statistics on the number of differential genes and GO pathway enrichment analysis in the SA-F vs. SA-M group. (**C**) Statistics on the number of differential genes and Go pathway enrichment analysis in the SA-F vs. GA-F group. (**D**) Statistics on the number of differential genes and GO pathway enrichment analysis in the SA-M vs. GA-M group. (Antennae from gregarious females, GA-F; antennae from gregarious males, GA-M; antennae from solitary females, SA-F; antennae from solitary males, SA-M).

We performed differential gene expression (DEG) analysis between each pair of samples. Relative to GA-F, GA-M exhibited 227 upregulated and 176 downregulated genes (Figure 3A). SA-M presented 51 upregulated and 268 downregulated genes compared to SA-F (Figure 3B). When comparing phases, GA-F exhibited 256 upregulated and 130 downregulated genes relative to SA-F (Figure 3C), whereas GA-M showed 580 upregulated and 187 downregulated genes relative to SA-M (Figure 3D).

An in-depth analysis of the DEGs was performed using Gene Ontology (GO) enrichment. We found that in GA-M antenna, the upregulated genes compared to GA-F were mainly associated with cellular processes, while the GO enrichment pathways of downregulated genes were primarily associated with oxidoreductase activity (Figure 3A). In SA-M, the GO enrichment pathways of upregulated genes compared to SA-F were mainly associated with olfactory receptor activity, whereas downregulated genes were primarily linked to organic cyclic compound binding (Figure 3B). In GA-F antenna, the GO enrichment pathways of upregulated genes compared to SA-F were mainly involved in defense response, while the downregulated genes were predominantly associated with amino acid transmembrane transport (Figure 3C). In GA-M, the GO enrichment pathways of upregulated genes compared to SA-M were mainly related to aminoglycan metabolic process, whereas downregulated genes were mainly involved in olfactory receptor activity. (Figure 3D and Table S4).

**Figure 4:**
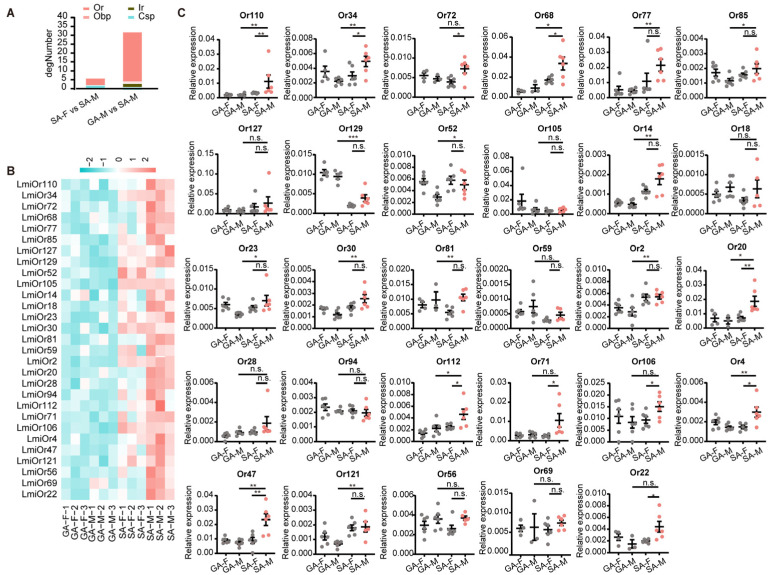
Antennal transcriptome profiling of Or genes abundantly expressed in solitary male antenna compared to GA-M and SA-F. (**A**) Number of differentially expressed genes of olfactory genes in the comparisons SA-F vs. SA-M and GA-M vs. SA-M. (**B**) Heat map of the mRNA expression levels indicating higher expression of Ors in SA-M than in SA-F and GA-M, based on the antennal transcriptome data. LogFC > 1 and FDR < 0.05. (**C**) mRNA expression levels of locust Ors abundantly expressed in male antennae compared with female antennae, determined by quantitative polymerase chain reaction (qPCR) (n = 6). Data are presented as mean ± s.e.m. *p* values refer to two-way ANOVA and Student’s *t*-tests or Mann–Whitney U tests (n.s., no significant difference. * *p* < 0.05, ** *p* < 0.01, *** *p* < 0.001).

Among the differentially expressed genes, we identified 27 candidate odorant receptor (Or) genes that were significantly more abundantly expressed in the SA-M compared to the GA-M, and 4 candidate Or genes were significantly more abundantly expressed in the SA-M compared to the SA-F (Figure 4A,B and Table S5). The RNA expression level of these genes was further confirmed by qPCR analysis. Based on the transcriptome and qPCR analyses of 29 candidate Or genes, twelve Or genes exhibited significant sex × phase interaction effects by two-way ANOVA (Table S6). The five Or genes showing the strongest interaction effects were *LmigOr47* (F_1,21_ = 11.84, *p =* 0.003), *LmigOr4* (F_1,20_ = 10.18, *p =* 0.005), *LmigOr30* (F_1,20_ = 9.12, *p =* 0.007), *LmigOr34* (F_1,19_ = 8.48, *p =* 0.009), and *LmigOr22* (F_1,20_ = 6.48, *p =* 0.022). Simple main effects analysis revealed that *LmigOr47*, *LmigOr4*, *LmigOr34*, and *LmigOr22* displayed a consistent male-biased expression pattern in the solitary phase, with males showing significantly higher expression than females, whereas no significant sexual dimorphism was detected in the gregarious phase. Notably, *LmigOr129* exhibited a unique reversed pattern, showing gregarious-biased expression with a significant interaction effect (F_1,18_ = 4.90, *p =* 0.040). Additionally, *LmigOr52* displayed a significant main effect of sex (F_1,20_ = 8.82, *p =* 0.008), contrasting with the male-biased pattern observed in most other genes, while *LmigOr2* showed overall solitary-phase upregulation regardless of sex (F_1,21_ = 5.84, *p =* 0.025). The other 15 Or genes showed no significant difference.

In the comparison between SA-M and GA-M, 17 Or genes showed significantly higher expression levels in SA-M than GA-M, consistent with the transcriptome results (Figure 4B). These genes included *LmigOr110*, *LmigOr34*, *LmigOr68*, *LmigOr77*, *LmigOr85*, *LmigOr129*, *LmigOr52*, *LmigOr14*, *LmigOr23*, *LmigOr30*, *LmigOr81*, *LmigOr2*, *LmigOr20*, *LmigOr112*, *LmigOr4*, *LmigOr47*, and *LmigOr121* (Figure 4C). Comparison between SA-M and SA-F identified 11 Or genes with higher expression in SA-M compared with SA-F, consistent with the transcriptomic results (Figure 4B). These comprised *LmigOr110*, *LmigOr34*, *LmigOr72*, *LmigOr68*, *LmigOr20*, *LmigOr112*, *LmigOr71*, *LmigOr106*, *LmigOr4*, *LmigOr47*, and *LmigOr22* (Figure 4C).

## 4. Discussion

In this study, we compared the number of different types of sensilla on the antennae of gregarious and solitary locusts, as well as their EAG responses to volatiles and antennal transcriptomic profiles. Our results reveal adaptive differences in the olfactory systems of locusts with distinct social behaviors. Solitary locusts exhibit higher peripheral olfactory sensitivity, alongside disproportionately larger antennal lobes for enhanced primary processing traits likely favored by selection for detecting sparse resources in low-density environments [29,30]. Gregarious locusts, conversely, show reduced peripheral sensitivity but invest more in central integration with 50% larger mushroom body primary calyces relative to the antennal lobe and 30% larger overall brains [30]. This peripheral-to-central reorganization supports the sophisticated processing of complex chemical cues, associative learning, and decision-making required for generalist foraging and swarm cohesion, where abundant resources diminish the need for extreme sensitivity but dense social environments demand enhanced signal extraction [31]. Thus, phase differences represent strategic neural investment trade-offs, with genetic differences in sensilla number and Or expression interfacing with centrally reorganized circuits to shape distinct behavioral strategies [29]. Overall, our findings highlight the interplay between genetic and environmental factors in shaping insect olfaction, with implications for understanding ecological adaptations and evolutionary trajectories.

We found that solitary males possessed a significantly higher total number of antennal sensilla, an advantage primarily driven by an increased number of sensilla basiconica. However, in the gregarious phase, no significant difference was observed in the total number of sensilla between males and females. These results are consistent with the findings of Greenwood and Chapman in *L. migratoria*, although they used only one insect in each category when counting the total number of sensilla [32]. Across all combinations, sensilla basiconica were the most abundant type, followed by sensilla coeloconica, whereas sensilla trichodea and sensilla chaetica were much less numerous. This distribution pattern further corroborates the main role of sensilla basiconica in locust olfactory communication. Ors tuned to aggregation pheromones, sex pheromones, and anticannibalistic pheromones are expressed on neurons housed in the basiconica sensilla [3,10,33]. Possessing more sensilla basiconica, the primary units for olfactory reception, enhances their ability to detect sex pheromones and conspecific volatiles, thereby achieving greater reproductive success. In contrast, the total number of sensilla in females does not change significantly between the solitary and gregarious phases. This may be attributed to a female reproductive strategy that places greater emphasis on foraging and selecting suitable oviposition sites, rather than on long-distance searching for males [34]. Consequently, the sensory system of females is less sensitive to changes in population density than that of males. But, the results differ from the conclusions of previous research on the desert locust, *S. gregaria* [28], indicating that the sensilla of *L. migratoria* exhibit species-specific density-dependent plasticity. This specific difference in *L. migratoria* could reflect the distinct reproductive pressures under the two phases. At low density, solitary males face the significant challenge of finding, locating, and guarding females, thus evolving enhanced olfactory capacity. The distribution and number of antennal sensilla are crucial for locating sporadically distributed food resources and mating [35,36].

Through antennal transcriptome analysis of gregarious and solitary locusts, we found that Or genes were more highly expressed in the antenna of solitary males. This may explain the behavioral differences between the two phases and their higher olfactory sensitivity to chemical signals. Studies on different species of insects have shown that the expression of Or genes can exhibit plasticity in response to constantly changing internal (physiological state, hormone levels, and olfactory experiences) and external (chemical signals, light, temperature, etc.) environmental factors [37,38,39]. The expression of Or genes in the antenna is influenced by olfactory environment, blood-feeding odorant molecules and mating status, respectively, in honeybees [40,41], mosquitoes [42], and fruit flies [43,44]. Collectively, these studies demonstrate that Or gene expression exhibits remarkable plasticity in response to various internal and environmental factors across diverse insect species. Our findings contribute to the growing evidence that behavioral differences between phases are closely associated with molecular mechanisms underlying variations in olfactory sensitivity. The species-specific characteristics of olfactory receptor families and their regulatory mechanisms highlight the complexity of insect olfactory systems and their adaptive significance in different ecological contexts.

Solitary male locusts exhibited a stronger EAG response to compounds across all combinations, though the magnitude of difference was modest compared to the substantial variation in antennal sensillum numbers. This discrepancy suggests that while increased sensilla density provides the structural basis for enhanced olfactory detection, the relationship between sensilla abundance and EAG amplitude is not strictly linear. Previous studies have also demonstrated a non-linear relationship between sensillum numbers in different antennal regions and electrophysiological responses [45]. Even when sensillum numbers differ between gregarious and solitary phases, receptor densities may reach saturation in key regions [46,47]. Insect antennae possess multiple types of sensilla, including trichoid sensilla, basiconic sensilla, and coeloconic sensilla, each with distinct functional specificity and redundancy. Although the overall EAG responses appear similar, individual sensilla may vary in specificity and sensitivity [48,49]. Different sensillum types may exhibit overlapping responsiveness to the same compound classes [50,51]. Consequently, morphological differences in sensilla between gregarious and solitary locusts reflect their distinct ecological adaptation strategies, while the similarity in EAG responses underscores the plasticity and adaptability of the insect olfactory system.

The enhanced EAG response of solitary males is likely due to the combined effects of increased antennal sensilla and higher expression levels of odorant receptor genes [52]. The relationship between antennal sensilla characteristics and olfactory sensitivity is complex and multifaceted. Evidence across diverse insect taxa, such as dung beetles [36], *Oecophylla smaragdina* [53], and *Clunio marinus* [54], shows that sensilla morphology, distribution and composition vary among species and sexes, correlating with their distinct behavioral and olfactory responses. At the molecular level, the expression levels of odorant receptor genes serve as critical determinants of olfactory responses [55,56,57]. Particularly in locusts, which exhibit highly specific and low-redundancy odorant detection, reducing the expression level of functional Ors would lead to decreased or completely abolished EAG responses [10,58]. Notably, the dose–response curves for compounds showed no saturation even at higher concentrations. This lack of saturation suggests that the insect olfactory system exhibits remarkable complexity and plasticity. EAG recordings reflect the integrated electrophysiological response of the entire antenna, involving the coordinated activity of multiple olfactory receptor neurons [59]. Some receptors may remain incompletely activated even at high concentrations [58,60]. Furthermore, each receptor exhibits distinct affinity and kinetic properties when compounds bind to different receptors. The physicochemical characteristics of the compounds themselves influence their saturation behavior—complex molecules may possess multiple binding sites [61].

The specific compounds eliciting strong EAG responses align with known ligands for locust Ors [11,13]. In our study, LmigOr68 and LmigOr20, which were confirmed to be tuned to phenol and cis-3-hexen-1-ol that elicited stronger EAG responses in solitary males, are upregulated in solitary males; this enhanced sensitivity likely stems from their elevated expression, facilitating the detection of both plant volatiles and conspecific pheromone components. Notably, this molecular specificity combines with increased sensillum numbers to create a synergistic effect that amplifies olfactory sensitivity. Locusts achieve functional optimization by modulating sensilla density rather than altering antennal morphology [4]. This contrasts sharply with the diverse and elaborate antennae of moths adapted for long-range pheromone detection [62], as well as the clubbed antennae of beetles utilized for coordinated host attacks [63]. Instead, locusts maintain simple filiform antennae, reflecting evolutionary conservatism likely due to their multimodal communication strategy integrating tactile, acoustic, and chemical signals, which obviates an exclusive reliance on olfaction [3,64,65]. Specifically, locusts exhibit substantial neural investment in the peripheral nervous system, where they possess 142 Ors [66], a number markedly exceeding that of moths [67]. In the central nervous system, locusts display more numerous olfactory glomeruli and sensory neurons underlying sensilla [68]. We hypothesize that this increased neural investment functions to lower odor detection thresholds for precise behavioral regulation, although the underlying evolutionary mechanisms warrant further investigation.

Future research can explore the developmental basis of these differences in response to density changes in locusts. For example, are there epigenetic mechanisms that can adjust the development of antennal sensilla or the expression of Or genes based on density conditions? Furthermore, studying the specific functions of different sensilla types in detecting different compounds and the role of supporting cells in chemical signal processing will also help deepen our understanding of the molecular mechanisms of locust olfaction.

## Data Availability

The RNA-seq data were deposited at the National Genomics Data Center under BioProject no. CRA037792.

## Supplementary Materials

The following supporting information can be downloaded at https://www.mdpi.com/article/10.3390/insects17030330/s1. Figure S1: Comparison of gene expression patterns between gregarious and solitary locusts of both sexes. Table S1: Primer used in qPCR analysis. Table S2: Statistical analysis of sex and phase effects on EAG responses to volatile compounds by two-way ANOVA. Table S3: Raw data and mapped statistics. Table S4: GO enrichment analysis of differentially expressed genes between male and female antennae. Table S5: Or genes were significantly more abundantly expressed in GA-M compared to SA-M and SA-M compared to SA-F. Table S6: Statistical analysis of sex and phase effects on candidate odorant receptor (Or) genes expression by two-way ANOVA.
