# Supplementary material for: Olfactory Susceptive Difference in Gregarious and Solitary Locusts
Source: Insects. 2026 Mar 18;17(3):330. doi: 10.3390/insects17030330 (PMC13027155; doi:10.3390/insects17030330)
Supplement: Supplementary file 1 [file insects-17-00330-s001.zip › insects-4152761-supplementary.pdf]

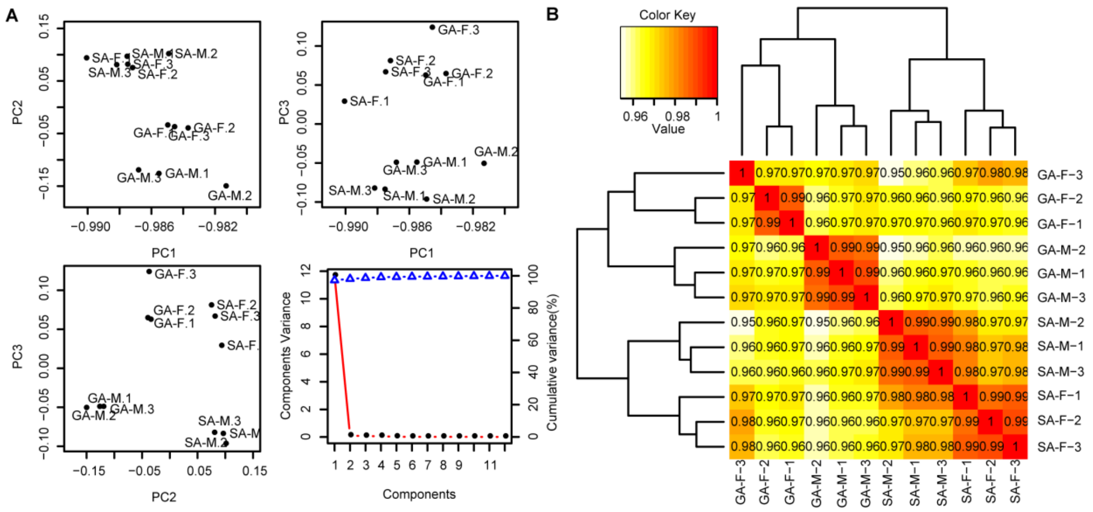

Figure S1. Overall transcriptome analysis of antenna from gregarious and solitary locusts of both sexes. (A) Principal component analysis (PCA) of all 12 samples from gregarious and solitary female and male locusts. (B) Cluster analysis of gene expression levels in GA-F, GA-M, SA-F and SA-M. (antennae from gregarious females, GA-F; antennae from gregarious males, GA-M; antennae from solitary females, SA-F; antennae from solitary males, SA-M)

Table S1. Primer used in qPCR analysis

Gene name	Forward primer	Reverse primer
LmiOr110	CTCACCTACTGGCTGATGTTCGC	GGGACACTCCAATCTTCCTCTTT
LmiOr34	CGATTCCAGGGCAGAACAAAGC	TCAAGATGGTGGGGATGATGGC
LmiOr72	ATCGGGGAAAAGGTATGGTAG	CAGATGCCTATTGCGAAAGTG
LmiOr68	CGCAGGAAGACGAGAAAAGG	CAACGCTCACGGCGAACT
LmiOr77	AGCGCCGTGTCGCTCTACTTCT	GCGTGGTACTGTCGCTGGTTGC
LmiOr85	CTGTACCAGTCGGTGACTCTGTA	TTGTCCTTATTTTCCTGTTCCTC
LmiOr127	GTCGCCTCCGCTGTCTGTCTGG	AAGGGCACGGTCATCACTGGGT
LmiOr129	GTCTCCACCCCGTTACTTGTCC	CCACCAGTACGCTGAAAGATTG
LmiOr52	GAGTTCTTCTTCCTGGCGGTCAT	GAGCTCCTCGTACACTGCATCA
LmiOr105	TTTCGCCTGCTTGATGACTGCT	TCCGCCAATGACTTCTTGCTCT
LmiOr14	GCTCATCCTACTGACGAGGCACC	CGATGGATTCGTTTTCTGATAGTG
LmiOr18	ACATCGTCTTCTGCGTCCACAT	GTCTCGGTCGAAGCAGATTTCA
LmiOr23	GCGGAGTTCCTGAGCTGGGAGG	GTCGTAGGGCAGCGCCAAGATG
LmiOr30	GAGCTGCTCAACCTGTCGTTCG	AATACAGTCCCGCAGTTCTTGG
LmiOr81	CTGTCCAGGGTGATCGTCTG	AGCATCGGGTAGTTGTGGC
LmiOr59	TACGCTATGCAGGTCGTTTCCA	AACTTCCTCGCCTTCATTTGAT
LmiOr2	GCCATACGTCTACACTGTTGTTTT	TCCTCATTTTGCCTTTGATTTG
LmiOr20	CAGCGACCTGGGCTACCT	ACAGCACGATGAACACGGA
LmiOr28	TTCCAGTATTAACTACCGTTCGAT	GCTCTTTGCCTGATATGTTTTGA
LmiOr94	GTCAGCATCTGCTTCCACATCA	CCGTCTGGTAAGCGTAGAGTGTC
LmiOr112	GGCGTTGCCGAAAGGCAGAGTG	TGATGATAATTTGATGGTGCTGTA

LmiOr71	ACAACAACAGCCACTTCTACGG	CACTTCCTGGTTCAACTTCAAAAT
LmiOr106	GTACGCTTTCCAGATGCTACTAAT	AGGTTGACTTCTTTCCCCGACT
LmiOr4	GGACTGGGAGACTCGTATGTTG	TTGCTGTAAATAGAAGACGACCA C
LmiOr47	CACTACACCCACAGCGAGACCT	AGAGCGGTACGATGCACCACAT
LmiOr121	CGATAGCAATCGTTGGCTGGAT	TGGTGAAGGACTGGAAGGCGTA
LmiOr56	ATGGACTCCGACTGTTTCTTCA	TCAGTGTCATCGACAGGTAGTGT
LmiOr69	CGGCTTGTCGTACTTTGCA	GGGATACTCGGCTTTCTCGT
LmiOr22	ATTGGTGCCTCTACTTGCTTCC	CCACGTTCTCGCTTTCTGTTATA

Table S2. Statistical analysis of sex and phase effects on EAG responses to volatile compounds by two-way ANOVA.

Compounds	Interaction F	Interaction P	Sex F	Sex P	Phase F	Phase P	Pattern
Dibutyl phthalate	59.07	<0.001	0.60	0.443	0.68	0.413	Interaction
4-Vinylanisole	0.15	0.697	7.21	0.010	5.46	0.024	Both main
Anisole	1.90	0.175	8.47	0.005	7.00	0.011	Both main
Veratrole	0.44	0.508	16.12	<0.001	0.07	0.785	Sex only
Benzaldehyde	0.54	0.466	9.91	0.003	7.97	0.007	Both main
2-penten-1-ol	0.01	0.933	7.63	0.008	0.04	0.849	Sex only
2-ethyl-1-hexanol	0.19	0.669	0.63	0.430	3.57	0.065	No effect
Pentanoic acid	0.23	0.631	0.58	0.449	11.22	0.002	Phase only
E-2-Hexenol	0.02	0.901	9.47	0.003	0.03	0.869	Sex only
Hexanal	0.07	0.800	0.63	0.433	0.68	0.414	No effect
Trans-2-Hexenal	0.02	0.882	2.95	0.092	0.08	0.777	No effect
Phenylacetonitrile	0.04	0.842	10.39	0.002	1.09	0.302	Sex only
Phenethyl alcohol	0.03	0.859	13.88	<0.001	0.29	0.591	Sex only
Guaiacol	0.21	0.653	12.04	0.001	0.68	0.412	Sex only
Cis-3-Hexen-1-ol	0.44	0.508	7.34	0.009	0.00	0.954	Sex only
Phenol	2.31	0.135	18.12	<0.001	21.10	<0.001	Both main

Table S3. Raw data and mapped statistics.

Sample	ReadLength	RawReads	#AfterFilter_PEreads	TotalMapped
GA-F-1	100_100	35372326	32426180	27782448
GA-F-2	100_100	33727884	31318372	26621165
GA-F-3	100_100	32785316	30045472	25912863
GA-M-1	100_100	34519776	31994764	27116176
GA-M-2	100_100	33753568	31529872	26732833
GA-M-3	100_100	36781080	33962640	28721071
SA-F-1	100_100	35645862	33096310	28533979
SA-F-2	100_100	32593738	29934138	25777684
SA-F-3	100_100	35037884	32371256	27829627
SA-M-1	100_100	32221090	29333522	24287974
SA-M-2	100_100	31713416	29163080	24518203
SA-M-3	100_100	30710012	28861784	24681163

Table S4. GO enrichment analysis of differentially expressed genes between male and female antennae.

GO_Term	GO_Class	x1	Pvalue
**GA-F-VS-GA-M.down**			
oxidoreductase activity	MF	20	4.00E-09
extracellular region	CC	7	4.25E-04
lipid transporter activity	MF	3	5.33E-04
oxidation-reduction process	BP	13	6.18E-04
lipid transport	BP	3	6.24E-04
lipid localization	BP	3	6.24E-04
binding	MF	32	1.54E-03
cell adhesion	BP	5	1.86E-03
biological adhesion	BP	5	1.86E-03
nucleic acid binding	MF	1	2.39E-03
**GA-F-VS-GA-M.up**			
cellular process	BP	12	2.51E-06
cellular metabolic process	BP	5	1.70E-05
cellular macromolecule metabolic process	BP	2	5.38E-05
cell	CC	5	1.43E-04
cell part	CC	5	1.43E-04
structural constituent of cuticle	MF	7	1.66E-04
intracellular	CC	5	2.80E-04
intracellular part	CC	4	6.22E-04
defense response	BP	3	6.87E-04
chitin binding	MF	5	1.13E-03
**SA-F-VS-SA-M.down**			
organic cyclic compound binding	MF	7	3.42E-07
heterocyclic compound binding	MF	7	3.57E-07
lipid transporter activity	MF	5	4.17E-06
lipid transport	BP	5	5.51E-06
lipid localization	BP	5	5.51E-06
peptidoglycan muralytic activity	MF	5	9.15E-06
oxidoreductase activity	MF	22	2.42E-05
cellular macromolecule metabolic process	BP	3	3.67E-05
defense response	BP	4	3.88E-05
nucleic acid binding	MF	1	5.08E-05
**SA-F-VS-SA-M.up**			
olfactory receptor activity	MF	4	4.88E-04
sensory perception of smell	BP	4	4.88E-04
defense response	BP	2	6.87E-04
odorant binding	MF	4	8.18E-04

sensory perception of chemical stimulus	BP	4	2.42E-03
sensory perception	BP	4	2.67E-03
neurological system process	BP	4	2.72E-03
system process	BP	4	2.76E-03
multicellular organismal process	BP	4	5.13E-03
1-acylglycerol-3-phosphate O-acyltransferase activity	MF	1	5.27E-03
**GA-F-VS-SA-F.down**			
defense response	BP	4	1.65E-05
aminoglycan catabolic process	BP	5	3.00E-05
carbohydrate derivative catabolic process	BP	5	5.94E-05
glycosaminoglycan metabolic process	BP	4	6.87E-05
response to stress	BP	8	2.00E-04
organonitrogen compound catabolic process	BP	5	3.40E-04
N-acetylmuramoyl-L-alanine amidase activity	MF	3	6.33E-04
peptidoglycan catabolic process	BP	3	6.33E-04
peptidoglycan metabolic process	BP	3	6.33E-04
glycosaminoglycan catabolic process	BP	3	6.33E-04
**GA-F-VS-SA-F.up**			
amino acid transmembrane transport	BP	2	1.51E-03
amino acid transmembrane transporter activity	MF	2	1.51E-03
amino acid transport	BP	2	1.51E-03
organic acid transport	BP	2	1.51E-03
organic acid transmembrane transport	BP	2	1.51E-03
carboxylic acid transmembrane transport	BP	2	1.51E-03
organic acid transmembrane transporter activity	MF	2	2.59E-03
carboxylic acid transmembrane transporter activity	MF	2	2.59E-03
heme binding	MF	5	3.01E-03
tetrapyrrole binding	MF	5	3.09E-03
**GA-M-VS-SA-M.down**			
aminoglycan metabolic process	BP	20	2.33E-12
aminoglycan catabolic process	BP	11	9.32E-10
defense response	BP	8	1.65E-09
carbohydrate derivative catabolic process	BP	11	4.83E-09
structural constituent of cuticle	MF	16	8.88E-09
carbohydrate derivative metabolic process	BP	22	1.83E-08
glycosaminoglycan metabolic process	BP	8	4.51E-08
cellular process	BP	41	4.72E-08
peptidoglycan muralytic activity	MF	8	6.91E-08
cell	CC	15	8.42E-08
**GA-M-VS-SA-M.up**			
olfactory receptor activity	MF	27	8.83E-27
sensory perception of smell	BP	27	8.83E-27
odorant binding	MF	28	1.72E-26

sensory perception	BP	28	1.70E-22
neurological system process	BP	28	1.93E-22
system process	BP	28	2.18E-22
sensory perception of chemical stimulus	BP	27	1.42E-21
transmembrane receptor activity	MF	31	1.92E-20
transmembrane signaling receptor activity	MF	30	2.15E-20
multicellular organismal process	BP	28	2.62E-20

Table S5. Or genes were significantly more abundantly expressed in GA-M compared to SA-M and SA-M compared to SA-F

GA-M vs SA-M up	SA-F vs SA-M up
LmigOr110	LmigOr68
LmigOr34	LmigOr69
LmigOr72	LmigOr22
LmigOr68	LmigOr20
LmigOr77	
LmigOr85	
LmigOr127	
LmigOr129	
LmigOr52	
LmigOr105	
LmigOr14	
LmigOr18	
LmigOr23	
LmigOr30	
LmigOr81	
LmigOr59	
LmigOr2	
LmigOr20	
LmigOr28	
LmigOr94	
LmigOr112	
LmigOr71	
LmigOr106	
LmigOr4	
LmigOr47	
LmigOr121	
LmigOr56	

Table S6. Statistical analysis of sex and phase effects on candidate odorant receptor (Or) genes expression by two-way ANOVA.

Gene	Interaction F	Interaction P	Sex F	Sex P	Phase F	Phase P	Pattern
LmigOr47	11.84	0.003	0.26	0.613	0.00	0.976	Interaction
LmigOr4	10.18	0.005	1.42	0.249	1.08	0.313	Interaction
LmigOr30	9.12	0.007	2.95	0.101	0.12	0.737	Interaction
LmigOr34	8.48	0.009	2.68	0.118	0.52	0.482	Interaction
LmigOr23	7.27	0.014	5.01	0.037	0.44	0.513	Interaction
LmigOr22	6.48	0.022	1.03	0.327	0.48	0.498	Interaction
LmigOr20	5.74	0.030	0.17	0.683	0.00	0.965	Interaction
LmigOr72	5.58	0.032	0.34	0.568	1.91	0.188	Interaction
LmigOr14	5.00	0.037	0.14	0.709	6.15	0.022	Interaction
LmigOr129	4.90	0.040	1.08	0.313	82.78	<0.001	Interaction
LmigOr85	4.77	0.041	3.06	0.096	0.23	0.638	Interaction
LmigOr71	4.51	0.046	0.01	0.906	0.02	0.902	Interaction
LmigOr52	2.20	0.153	8.82	0.008	0.07	0.799	Sex only
LmigOr2	0.55	0.467	0.87	0.361	5.84	0.025	Phase only
LmigOr106	3.25	0.086	0.61	0.443	0.22	0.642	No effect
LmigOr110	3.01	0.098	0.00	0.990	0.15	0.699	No effect
LmigOr77	2.62	0.121	0.03	0.875	1.24	0.278	No effect
LmigOr68	2.11	0.167	0.20	0.658	3.34	0.088	No effect
LmigOr105	1.76	0.200	2.83	0.109	3.47	0.078	No effect
LmigOr81	1.73	0.208	0.69	0.420	2.17	0.161	No effect
LmigOr112	1.55	0.227	1.17	0.293	1.93	0.180	No effect
LmigOr121	1.54	0.230	2.52	0.129	3.66	0.071	No effect
LmigOr28	0.50	0.488	0.45	0.509	0.65	0.431	No effect
Lmig Or127	0.49	0.494	0.05	0.833	0.36	0.554	No effect
LmigOr56	0.38	0.547	1.46	0.241	0.49	0.493	No effect
LmigOr69	0.27	0.608	0.02	0.896	0.03	0.859	No effect
LmigOr18	0.19	0.666	1.12	0.303	0.77	0.390	No effect
LmigOr94	0.11	0.746	1.04	0.319	0.97	0.336	No effect
LmigOr59	0.00	0.982	0.96	0.338	2.89	0.106	No effect
